# Dynamic Oxidative States: Interplay of Aging, Metabolic Stress, and Circadian Rhythms in Modulating Stroke Severity

**DOI:** 10.3390/antiox15010054

**Published:** 2025-12-31

**Authors:** Jui-Ming Sun, Jing-Shiun Jan, Cheng-Ta Hsieh, Rajeev Taliyan, Chih-Hao Yang, Ruei-Dun Teng, Ting-Lin Yen

**Affiliations:** 1 Section of Neurosurgery, Department of Surgery, Ditmanson Medical Foundation, Chia-Yi Christian Hospital, Chia-Yi 600, Taiwan; 07178@cych.org.tw; 2 Department of Nursing, School of Nursing, Fooyin University, Kaohsiung 831, Taiwan; 3 Department of Pharmacology, School of Medicine, College of Medicine, Taipei Medical University, Taipei 110, Taiwan; d119101004@tmu.edu.tw (J.-S.J.); chyang@tmu.edu.tw (C.-H.Y.); 4 Division of Neurosurgery, Department of Surgery, Cathay General Hospital, Taipei 106, Taiwan; nogor@mail2000.com.tw; 5 School of Medicine, National Tsing Hua University, Hsinchu 300, Taiwan; 6 Department of Medicine, School of Medicine, Fu Jen Catholic University, New Taipei 242, Taiwan; 7 Neuropsychopharmacology Division, Department of Pharmacy, Birla Institute of Technology and Science-Pilani, Pilani Campus, Pilani 333031, India; taliyanraja@gmail.com; 8 Research Center for Neuroscience, Taipei Medical University, Taipei 110, Taiwan; 9 Department of Medical Research, Taipei Medical University Hospital, Taipei 110, Taiwan; 10 Department of Medical Research, Cathay General Hospital, Taipei 106, Taiwan

**Keywords:** oxidative stress, redox homeostasis, stroke, aging, metabolic stress, circadian rhythm, NRF2, SIRT1, AMPK, mitochondrial dysfunction

## Abstract

Oxidative stress is a defining feature of stroke pathology, but the magnitude, timing and impact of redox imbalance are not static. Emerging evidence indicates that physiological contexts, such as aging, metabolic stress, and circadian disruption, continuously reshape oxidative status and determine the brain’s vulnerability to ischemic and reperfusion injury. This review integrates recent insights into how these intrinsic modulators govern the transition from adaptive physiological redox signaling to pathological oxidative stress during stroke. Aging compromises mitochondrial quality control and blunts NRF2-driven antioxidant responses, heightening susceptibility to ROS-driven damage. Metabolic dysfunction, as seen in obesity and diabetes, amplifies oxidative burden through NADPH oxidase activation, lipid peroxidation, and impaired glutathione recycling, further aggravating post-ischemic inflammation. Circadian misalignment, meanwhile, disrupts the rhythmic expression of antioxidant enzymes and metabolic regulators such as BMAL1, REV-ERBα, and SIRT1, constricting the brain’s temporal window of resilience. We highlight convergent signaling hubs, NRF2/KEAP1, SIRT–PGC1α, and AMPK pathways, as integrators of these physiological inputs that collectively calibrate redox homeostasis. Recognizing oxidative stress as a dynamic, context-dependent process reframes it from a static pathological state to a dynamic outcome of systemic and temporal imbalance, offering new opportunities for time-sensitive and metabolism-informed redox interventions in stroke.

## 1. Introduction

Oxidative stress is a fundamental driver of ischemic brain injury, contributing to neuronal death, inflammation, and vascular dysfunction [1,2]. However, the degree of oxidative damage observed after stroke is strikingly variable, even under comparable ischemic conditions. Such variability challenges the traditional view of oxidative stress as a static byproduct of ischemia–reperfusion injury. Instead, accumulating evidence suggests that oxidative stress represents a dynamic physiological state, one that fluctuates in response to intrinsic factors such as aging, metabolic health, and circadian rhythm [3,4]. These physiological contexts shape the brain’s redox landscape before an insult occurs, determining its capacity to tolerate or succumb to oxidative injury [5,6]. Recognizing oxidative stress as a modifiable physiological determinant rather than a passive consequence reframes our understanding of stroke pathophysiology.

In this review, the term “stroke” refers primarily to ischemic stroke, which accounts for the majority of clinical cases and is characterized by cerebral hypoperfusion followed by reperfusion-associated oxidative injury. Although hemorrhagic stroke also involves oxidative and inflammatory processes, its initiating mechanisms and temporal dynamics differ substantially and are not the primary focus of this article.

In the healthy brain, reactive oxygen species (ROS) are not inherently deleterious; they act as essential messengers for cellular signaling, synaptic plasticity, and vascular tone. The brain’s redox homeostasis depends on a delicate balance between ROS generation and antioxidant defense systems, such as the glutathione, thioredoxin, and peroxiredoxin networks [7]. This balance, however, is continuously influenced by the organism’s metabolic and temporal state [8,9]. During aging, mitochondrial inefficiency, diminished mitophagy, and reduced NRF2-mediated transcriptional responses lead to a decline in redox resilience [10]. In metabolic disorders such as obesity and diabetes, chronic nutrient overload activates NADPH oxidases and perturbs the NADPH/GSH ratio, resulting in sustained oxidative load and endothelial dysfunction [8]. Circadian regulation adds yet another layer of complexity: antioxidant enzyme expression and mitochondrial activity oscillate across the 24 h cycle under the control of core clock components like BMAL1, CRY1, and REV-ERBα [11,12,13]. Disruption of these rhythms through aging, sleep deprivation, or metabolic imbalance desynchronizes redox defense from energy metabolism, narrowing the window of neuroprotection.

These converging influences reveal that the oxidative state of the brain is far from constant; it dynamically shifts with age, nutritional status, and time of day. Consequently, the severity of ischemic injury is not solely determined by the extent of oxygen deprivation but by when and in what physiological state it occurs. Recent studies have shown that stroke outcomes differ across circadian phases [14,15], that aged animals exhibit greater infarct volumes [16,17], and that metabolic syndrome amplifies oxidative and inflammatory cascades [18,19]. Together, these findings support the emerging concept that stroke severity is a function of dynamic oxidative modulation.

**Figure 1:**
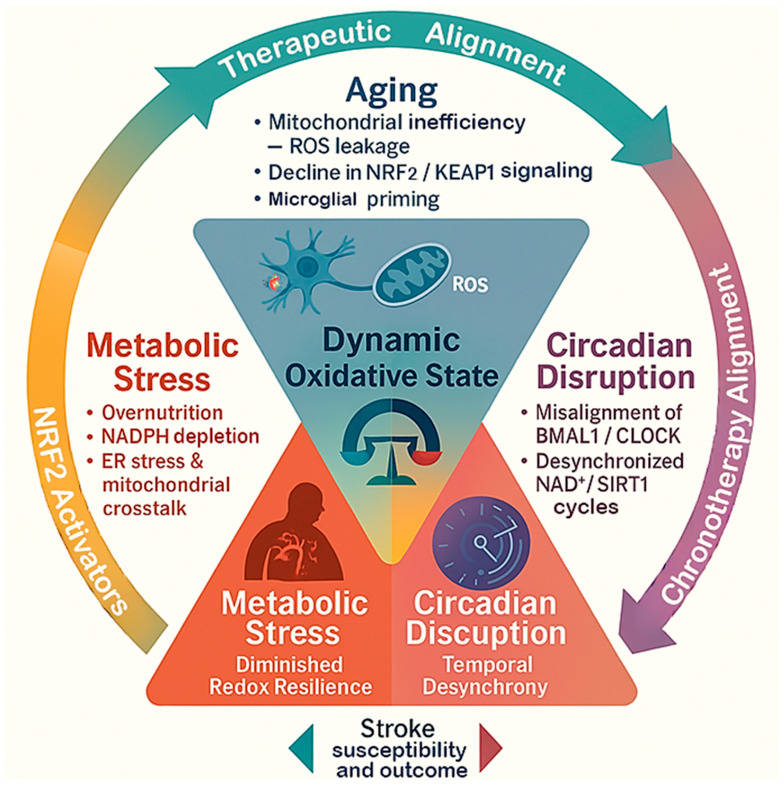
**The triangular framework of dynamic oxidative states in stroke.** This diagram shows how aging, metabolic stress, and circadian disruption interact to influence the brain’s oxidative state and shape stroke outcomes. Each factor contributes differently, aging reduces antioxidant capacity, metabolic stress increases oxidative load, and circadian disruption disturbs redox timing. Together, they determine the brain’s overall resilience or vulnerability to ischemic injury.

This review aims to introduce the concept of “dynamic oxidative states,” integrating evidence from aging biology, metabolic redox regulation, and circadian chronobiology to explain how physiological contexts predefine oxidative tone and influence ischemic vulnerability (Figure 1). We emphasize that these systems converge on shared molecular hubs, such as the NRF2/KEAP1 axis, the SIRT–PGC1α–AMPK signaling network, and the BMAL1–REV-ERBα clock components, which collectively calibrate redox balance and energy metabolism. Understanding these intersections provides a mechanistic framework for the variable oxidative responses observed across individuals and conditions. Overall, this perspective repositions oxidative stress from a secondary outcome of stroke to a preconditioning variable shaped by physiological rhythms and metabolic tone. By synthesizing these dimensions into a unified model, we aim to highlight how fluctuations in redox homeostasis contribute to stroke heterogeneity and to identify opportunities for interventions that restore metabolic and temporal alignment to enhance neuroprotection.

## 2. Diminished Redox Resilience in Aging

Aging is one of the strongest physiological determinants of oxidative vulnerability in the brain. With advancing age, the equilibrium between ROS generation and antioxidant defense progressively deteriorates, producing a basal pro-oxidative state even in the absence of pathology [20]. This chronic imbalance establishes a redox environment that predisposes the aged brain to ischemic injury, amplifying neuronal death, vascular dysfunction, and post-stroke inflammation [17,21]. The erosion of redox resilience arises from interconnected deficits in mitochondrial quality control, antioxidant transcriptional regulation, and metabolic coordination.

### 2.1. Mitochondrial Dysfunction and Impaired Quality Control

Mitochondria are both the principal source and target of ROS. Aging leads to cumulative oxidative damage to mitochondrial DNA (mtDNA) and proteins of the electron-transport chain, especially complexes I and III, which increases electron leakage and ROS production. This inefficiency establishes a self-propagating cycle in which ROS-induced mitochondrial injury further accelerates ROS generation [22,23]. In parallel, mitophagy becomes progressively defective due to attenuation of the PINK1/Parkin pathway and lysosomal decline, allowing dysfunctional mitochondria to persist [24]. These damaged organelles disturb calcium buffering, decrease ATP synthesis, and heighten susceptibility to ischemic depolarization [25,26]. Aged rodents consistently show exaggerated mitochondrial ROS bursts and larger infarct volumes after ischemic challenge [17,27], underscoring mitochondrial fragility as a primary driver of redox decline.

### 2.2. Attenuation of the NRF2-KEAP1 Antioxidant Pathway

The NRF2-KEAP1 axis serves as a master regulator of the inducible antioxidant defense system that safeguards cellular redox balance. Under oxidative or electrophilic stress, NRF2 dissociates from its cytoplasmic repressor KEAP1, translocates to the nucleus, and activates transcription of cytoprotective genes, including heme oxygenase-1 (HO-1), NAD(P)H quinone dehydrogenase 1 (NQO1), and enzymes involved in glutathione (GSH) synthesis [28]. Aging disrupts this adaptive response: nuclear NRF2 accumulation is diminished, KEAP1 expression increases, and the transcriptional activation of downstream antioxidant targets becomes blunted [29,30]. As a result, aged neurons exhibit delayed detoxification kinetics and sustained oxidative burden during ischemia–reperfusion episodes. Pharmacological NRF2 activators or genetic stabilization of NRF2 in aged models restore antioxidant capacity, mitigate ferroptosis, and reinforce blood–brain barrier (BBB) integrity, collectively limiting neuronal injury. Preclinical studies demonstrate that NRF2 reactivation attenuates inflammatory cascades and improves both histopathological and behavioral outcomes after stroke [31,32], underscoring NRF2 insufficiency as one important driver of redox vulnerability in the aging brain.

### 2.3. Decline in Sirtuin-Dependent Metabolic Regulation

Sirtuins, particularly SIRT1 and SIRT3, link NAD^+^ metabolism to antioxidant defense. With age, intracellular NAD^+^ pools diminish, leading to hypo-activation of these deacetylases. Reduced SIRT3 activity compromises deacetylation of mitochondrial superoxide dismutase 2 (SOD2) and PGC-1α, lowering oxidative-phosphorylation efficiency and ROS clearance [33]. Meanwhile, loss of SIRT1 activity disrupts the transcriptional control of antioxidant genes mediated by FOXO3 and PGC-1α, further uncoupling nuclear-mitochondrial coordination [29,34]. Restoration of NAD^+^ pools through supplementation with precursors such as nicotinamide riboside (NR) or nicotinamide mononucleotide (NMN) reactivates Sirtuin signaling, promotes mitochondrial biogenesis, and improves ischemic outcomes in aged models [35]. Post-ischemic administration of NMN or NR replenishes NAD^+^, elevates SIRT1 and SIRT3 activity, normalizes mitochondrial protein acetylation, restores SOD2 function, reduces ROS generation, and prevents mitochondrial fragmentation, resulting in smaller infarct volumes and improved neurological outcomes in aged rodents [36]. These findings highlight the NAD^+^-SIRT-PGC-1α regulatory axis as a central node of the age-related metabolic and redox decline, linking energy sensing to antioxidant resilience during ischemic stress.

### 2.4. Loss of Proteostasis and Exacerbated Neuroinflammation

Age-related disruption of proteostasis and persistent neuroinflammatory activation jointly exacerbate oxidative stress and neuronal vulnerability during ischemia–reperfusion. With advancing age, the efficiency of both the ubiquitin-proteasome and autophagy-lysosome degradation systems declines, resulting in the accumulation of oxidized, misfolded, and aggregated proteins [37]. These dysfunctional proteins not only impair mitochondrial and cytosolic functions but also generate additional ROS, thereby fueling a self-perpetuating oxidative cycle [38]. During reperfusion, increased protein oxidation and aggregation further accelerate neuronal degeneration [39], particularly in highly vulnerable regions such as the hippocampal CA1.

Compromised autophagic flux in the aged brain restricts the clearance of damaged organelles and protein aggregates, amplifying oxidative burden and energy stress. Ineffective degradation processes also promote the buildup of lipofuscin and advanced glycation end-products (AGEs), which act as chronic intracellular ROS sources and extracellular inflammatory cues [40,41]. Concurrently, microglia exhibit a primed phenotype characterized by exaggerated activation and hypersecretion of pro-inflammatory mediators, including ROS, reactive nitrogen species (RNS), TNF-α, and IL-1β, upon ischemic challenge [1,42]. Together, these maladaptive responses establish a feed-forward loop linking impaired proteostasis, sustained oxidative stress, and overactive neuroinflammation, ultimately driving irreversible neuronal damage and redox inflexibility in the aging brain.

### 2.5. Integrated Consequences for Stroke Pathophysiology in Aging

The convergence of mitochondrial inefficiency, attenuated NRF2 signaling, sirtuin decline, and chronic neuroinflammatory priming creates a maladaptive redox environment characterized by elevated basal ROS and diminished adaptive capacity [43,44]. During ischemic insult, this compromised reserve fails to mount an adequate antioxidant response, resulting in uncontrolled oxidative damage, neuronal death, and impaired post-stroke recovery [45]. Clinically, aging is associated with larger infarct volumes, delayed neurological improvement, and reduced responsiveness to antioxidant or neuroprotective interventions [46,47,48].

**Figure 2:**
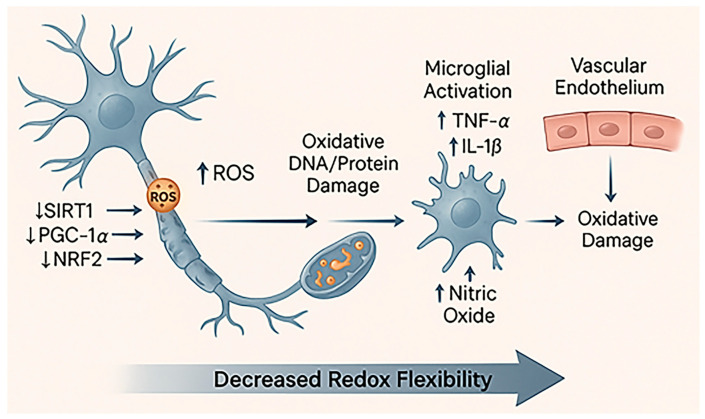
**Diminished redox resilience in aging.** This schematic illustrates how aging weakens neuronal and vascular redox defense. Progressive mitochondrial damage leads to excessive ROS production and reduced activity of key regulators such as SIRT1, PGC-1α, and NRF2, resulting in lower antioxidant gene expression [51]. Accumulated oxidative damage triggers microglial activation (increase in TNF-α and IL-1β) and endothelial dysfunction, characterized by decreased nitric oxide (NO) availability and impaired vascular tone. Together, these changes reduce the brain’s redox flexibility, leaving neurons more vulnerable to oxidative and ischemic stress.

Importantly, many pathways that deteriorate with age, including NRF2, SIRT1, and PGC-1α, are also tightly regulated by metabolic and circadian cues, linking redox homeostasis to systemic physiology [49,50]. This integrative framework underscores that redox failure in the aged brain is not an isolated event but reflects the breakdown of coordinated metabolic, transcriptional, and circadian control (Figure 2). Consequently, aging converts oxidative stress from a transient signal into a persistent pathological driver, one that determines stroke severity and recovery through the dynamic state of redox and metabolic regulation at the time of injury.

## 3. Oxidative Overload Under Metabolic Stress and Obesity

Metabolic stress, particularly in the context of obesity and diabetes, represents a chronic systemic driver of oxidative imbalance that predisposes the brain to vascular and neuronal injury [52]. Unlike aging, which gradually diminishes redox adaptability, metabolic stress imposes a state of persistent oxidative overload stemming from nutrient excess, lipid peroxidation, and altered energy metabolism [53,54,55]. These metabolic disturbances weaken endogenous antioxidant defenses while priming inflammatory and endothelial responses that exacerbate ischemic damage [56,57]. The resulting metabolic-redox imbalance not only amplifies stroke severity but also impedes post-ischemic recovery [45], highlighting the pathological synergy between metabolic dysfunction and oxidative stress.

### 3.1. NADPH Oxidase Activation and Vascular Oxidative Burden

One of the most prominent sources of ROS under metabolic stress is the NADPH oxidase (NOX) family, particularly NOX2 and NOX4, which are upregulated in vascular endothelial cells, adipocytes, and immune cells in obesity [58,59,60]. Chronic nutrient excess, notably elevated glucose and free fatty acids, elevates intracellular diacylglycerol (DAG) levels, activating protein kinase C (PKC) [61]. PKC phosphorylates cytosolic NOX subunits such as p^47^phox, promoting their translocation and assembly with membrane-bound components to form the active enzyme complex [62]. Concurrently, PKC-mediated activation of the small GTPase Rac1 enhances NOX complex stabilization at the membrane, further driving superoxide production [63].

In cerebral vasculature, excessive NOX activation depletes NO bioavailability, leading to endothelial dysfunction characterized by vasoconstriction, increased permeability, and microvascular rarefaction [64]. Experimental models of obesity and type 2 diabetes demonstrate elevated NOX4 expression in the neurovascular unit, which correlates with impaired cerebral perfusion and enlarged infarct volumes following ischemia [65]. Pharmacological inhibition or genetic suppression of NOX4 significantly ameliorates these effects [66,67], underscoring NOX-derived ROS as a principal mediator of metabolic oxidative stress and vascular vulnerability in the brain.

### 3.2. Lipid Peroxidation and Reactive Aldehyde Accumulation

Excess dietary fat and impaired lipid metabolism increase susceptibility to lipid peroxidation, generating reactive aldehydes such as 4-hydroxynonenal (4-HNE) and malondialdehyde (MDA) [68]. These electrophilic molecules covalently modify proteins and nucleic acids, forming adducts that disrupt mitochondrial enzymes, ion channels, and cytoskeletal integrity [69]. The accumulation of lipid peroxidation products is particularly deleterious in the ischemic brain, where oxygen reintroduction during reperfusion accelerates oxidative chain reactions within polyunsaturated lipid membranes. In obese animals, elevated 4-HNE–protein adducts are observed in the cortex and hippocampus, accompanied by mitochondrial swelling and neuronal apoptosis [70,71]. Beyond direct oxidative toxicity, lipid peroxidation products activate stress-responsive signaling pathways, including NF-κB and inflammasome cascades, thereby propagating inflammation throughout the neurovascular microenvironment [72]. The chronic accumulation of reactive aldehydes reinforces oxidative stress, disrupts mitochondrial and autophagic quality control, and sustains a self-amplifying cycle of neuroinflammation and neuronal degeneration in the ischemic brain [73].

### 3.3. Impairment of the Glutathione and NADPH Redox Systems

The GSH system constitutes the major intracellular antioxidant defense, responsible for neutralizing ROS and detoxifying lipid peroxides. Metabolic overload and obesity disrupt this system through several convergent mechanisms. Chronic hyperglycemia depletes NADPH reserves via activation of the polyol and hexosamine pathways, while excessive fatty acid oxidation consumes reducing equivalents and generates ROS at rates that exceed detoxification capacity [74]. As a result, the GSH/GSSG ratio declines, reflecting a shift toward an oxidized cellular state [75].

Furthermore, obesity-induced insulin resistance suppresses the expression of glutamate–cysteine ligase, the rate-limiting enzyme for GSH synthesis [76]. This depletion of redox buffering capacity compromises neuronal and endothelial resilience against ischemic oxidative surges.

In clinical studies, patients with metabolic syndrome exhibit reduced GSH levels and elevated GSSG, which correlate with increased vascular permeability, microvascular rarefaction, and poorer post-stroke outcomes [77,78]. Consistently, experimental models of insulin resistance and obesity demonstrate that GSH depletion leads to accumulation of oxidative damage markers such as 4-HNE and MDA, disruption of endothelial barrier integrity, and enlargement of infarct volume following cerebral ischemia [79,80]. Restoration of GSH levels, either through precursor supplementation or pharmacological enhancement of glutamate-cysteine ligase activity, ameliorates endothelial dysfunction and reduces ischemic injury in these models [79].

### 3.4. Endoplasmic Reticulum Stress and Mitochondrial Cross-Talk

Persistent nutrient excess triggers endoplasmic reticulum (ER) stress, contributing to oxidative overload through protein misfolding and calcium dysregulation [81]. The unfolded protein response (UPR) initially serves an adaptive role but becomes maladaptive under chronic metabolic burden, activating C/EBP homologous protein (CHOP) and c-Jun N-terminal kinase (JNK)-dependent apoptotic signaling [82,83]. Enhanced ER-mitochondria tethering facilitates excessive calcium flux into mitochondria, stimulating ROS generation and mitochondrial permeability transition [84]. This bidirectional amplification between ER stress and mitochondrial dysfunction establishes a pathological feedback loop that sustains oxidative stress in obesity-associated conditions [85]. In the context of cerebral ischemia, this pre-existing ER-mitochondrial coupling exacerbates neuronal apoptosis and compromises reperfusion recovery [86,87], thereby linking systemic metabolic stress to heightened redox vulnerability in the brain [88].

### 3.5. Neuroinflammatory and Endothelial Consequences

Beyond intracellular oxidative disturbances, metabolic stress profoundly reshapes systemic and neurovascular inflammation. Obese adipose tissue releases proinflammatory adipokines, including TNF-α, IL-6, and leptin, while reducing protective mediators such as adiponectin [89,90]. These cytokines activate endothelial NADPH oxidase complexes and promote leukocyte adhesion to the vascular endothelium, driving endothelial activation and platelet recruitment [91]. The ensuing microvascular inflammation increases blood–brain barrier permeability and fosters microthrombus formation, conditions that amplify ischemic injury severity. Concurrently, macrophages and microglia exposed to lipid excess adopt a pro-oxidant, M1-like phenotype marked by NOX2 activation and impaired mitochondrial respiration [92,93]. This sustained inflammatory-oxidative interplay perpetuates ROS generation and exacerbates secondary tissue damage following reperfusion [94]. Collectively, these mechanisms establish metabolic inflammation as a key amplifier of neurovascular vulnerability under ischemic stress.

### 3.6. Integration with Stroke Pathophysiology Under Metabolic Stress

**Figure 3:**
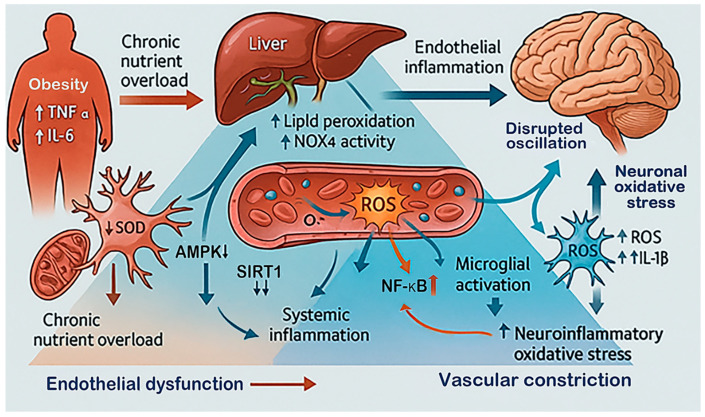
**Sustained oxidative load in metabolic stress and obesity.** This diagram illustrates how chronic metabolic stress and obesity create a persistent oxidative environment that affects the brain. Excess nutrients and inflammation from adipose tissue (elevated TNF-α, IL-6 and free fatty acids) and the liver (increase in NOX4 activity and lipid peroxidation) increase systemic ROS. These changes cause endothelial dysfunction, reducing vascular flexibility and oxygen delivery to the brain [90,97]. In response, microglia become overactivated, and neurons experience oxidative damage. Disturbances in signaling pathways, such as reduced AMPK and SIRT1 activity alongside enhanced NF-κB signaling, sustain redox imbalance and increase susceptibility to ischemic damage [98].

The metabolic–oxidative interactions outlined above converge to produce a state of chronic redox overload that compromises vascular integrity and neuronal resilience long before an ischemic event occurs. When stroke does happen, this pre-existing imbalance amplifies oxidative bursts, endothelial dysfunction, and immune cell activation, resulting in more extensive tissue injury. In clinical observations, patients with metabolic syndrome or type 2 diabetes exhibit higher oxidative biomarkers, larger infarcts, and poorer responses to thrombolytic or antioxidant therapies [45,52]. Molecular mediators of metabolic oxidative stress, including NOX4, SIRT1, and AMPK [95,96], overlap with those influenced by aging and circadian disruption, underscoring a shared redox regulatory network across physiological contexts. Metabolic stress thus establishes a persistent biochemical environment marked by sustained ROS generation, impaired antioxidant regeneration, and exaggerated inflammatory reactivity. Through NOX activation, lipid peroxidation, and GSH depletion, obesity and related disorders transform oxidative stress from a transient signal into a chronic systemic burden that predisposes the brain to ischemic injury (Figure 3). Recognizing this metabolic–redox coupling highlights the need for therapeutic approaches that integrate metabolic correction with redox-targeted neuroprotection.

## 4. Temporal Control of Redox Homeostasis by Circadian Rhythms

Beyond age and metabolic status, circadian rhythms provide an additional temporal layer of regulation that dynamically modulates oxidative balance and ischemic vulnerability. The brain’s intrinsic molecular clock, composed of transcriptional–translational feedback loops, synchronizes metabolic, inflammatory, and antioxidant processes to the 24 h light–dark cycle. Disruption of this rhythm, whether by genetic, environmental, or behavioral factors, impairs redox coordination and increases susceptibility to oxidative injury. Consequently, circadian regulation represents not only a determinant of physiological resilience but also a promising target for stroke prevention and chronotherapeutic intervention.

### 4.1. Molecular Architecture of the Circadian Clock

The circadian clock operates through interlocked feedback loops centered on the BMAL1/CLOCK transcriptional complex and its repressors, PER (Period) and CRY (Cryptochrome) proteins [99]. BMAL1 and CLOCK heterodimerize to drive the rhythmic transcription of Per and Cry genes, whose protein products inhibit BMAL1/CLOCK activity, forming a 24 h oscillation. Parallel stabilizing loops involve REV-ERBα/β and RORα/β, which, respectively, repress or activate Bmal1 transcription, maintaining rhythmic balance [100]. These molecular oscillations extend beyond the suprachiasmatic nucleus (SCN) into peripheral tissues, including the vasculature, liver, and glia, aligning redox metabolism with daily activity patterns. Many antioxidant enzymes, including SOD, glutathione peroxidase (GPx), and catalase, exhibit circadian oscillations, reflecting clock-dependent transcriptional control [101,102].

### 4.2. Circadian Regulation of Antioxidant Defense

The circadian clock exerts direct control over cellular redox balance by orchestrating rhythmic expression of antioxidant systems. During the active phase, BMAL1/CLOCK complexes enhance NRF2 signaling and upregulate downstream antioxidant genes such as HO-1, NQO1, and GCLM, aligning peak detoxification capacity with elevated metabolic activity and ROS production [12]. Conversely, in the rest phase, reduced NRF2 activity and increased REV-ERB expression suppress antioxidant transcription, permitting transient ROS accumulation that serves physiological redox signaling [11,103,104]. This oscillatory rhythm ensures cyclic restoration of redox homeostasis and prevents sustained oxidative burden.

Disruption of Bmal1 or Cry1/2 expression abolishes these redox oscillations, resulting in persistent oxidative stress, mitochondrial fragmentation, and features of premature neuronal aging [11]. In the brain, loss of circadian control over antioxidant enzymes heightens vulnerability to ischemic injury, as demonstrated by increased infarct volumes and poorer neurological recovery in clock-deficient mice [13,105]. These findings underscore the circadian clock as a temporal coordinator of antioxidant defense and a determinant of neurovascular resilience.

### 4.3. Clock-Metabolism Coupling and Mitochondrial Function

Circadian oscillations extend deeply into mitochondrial energetics and NAD^+^ metabolism, two pivotal determinants of cellular redox homeostasis. SIRT1, an NAD^+^-dependent deacetylase, interacts with CLOCK and BMAL1 to modulate transcriptional amplitude and maintain rhythmic coherence [106,107]. NAD^+^ levels themselves exhibit circadian oscillation, governed by rhythmic expression of Nampt, the rate-limiting enzyme in the NAD^+^ salvage pathway [108]. This establishes a bidirectional feedback loop wherein cellular redox status modulates clock precision, while circadian cues orchestrate mitochondrial biogenesis, oxidative phosphorylation, and ROS detoxification.

Disruption of this clock-metabolism coupling, as seen in aging and metabolic syndrome, diminishes NAD^+^ availability and SIRT1 activity, leading to impaired mitochondrial function and excessive ROS accumulation [106,109]. Conversely, restoration of circadian NAD^+^ rhythms, via timed feeding, caloric restriction, or supplementation with NAD^+^ precursors such as nicotinamide riboside or nicotinamide mononucleotide, enhances SIRT1 activation, reinforces antioxidant defense, and improves ischemic resilience [110,111].

### 4.4. Temporal Variation in Stroke Severity and Outcomes

Both experimental and clinical evidence demonstrate that the timing of stroke onset profoundly influences ischemic severity and recovery [112]. In rodents, ischemia occurring during the rest phase results in larger infarct volumes, elevated oxidative stress, and worsened neurological outcomes compared with injury during the active phase [113]. In humans, whose circadian phases are inverted, nighttime strokes similarly correlate with poorer prognosis and delayed functional recovery [15].

This phase-dependent vulnerability mirrors circadian oscillations in antioxidant defense, mitochondrial efficiency, and vascular dynamics [114]. Endothelial NO production and cerebral blood flow peak during the active phase, supporting optimal perfusion and redox control [115]. By contrast, during the rest phase, diminished antioxidant enzyme activity and heightened inflammatory responsiveness render neural tissues more susceptible to oxidative injury [116]. Together, these observations highlight circadian phase alignment as a critical, yet often overlooked, determinant of ischemic resilience and recovery potential.

### 4.5. Circadian Disruption and Ischemic Vulnerability

Modern lifestyle factors, such as irregular sleep schedules, shift work, and exposure to artificial light at night, chronically disturb circadian organization. Such disruption desynchronizes the central pacemaker in the SCN from peripheral oscillators, leading to temporal uncoupling between metabolic activity and antioxidant readiness [117,118]. In animal models, chronic circadian misalignment heightens vascular oxidative stress, promotes NOX-dependent ROS production, and impairs endothelial repair capacity [119,120].

Similarly, genetic ablation of clock components such as Bmal1 or Per2 reproduces these pathological features, resulting in exaggerated microglial activation, mitochondrial oxidative damage, and neuronal vulnerability [121]. These alterations parallel the cellular phenotypes of aged or metabolically stressed brains, suggesting that circadian dysregulation serves as a temporal amplifier of oxidative pathology [122]. By bridging environmental perturbations with molecular redox imbalance, circadian disruption transforms transient metabolic stress into sustained ischemic susceptibility.

### 4.6. Chronotherapy and Redox Modulation

The temporal nature of redox regulation offers opportunities for chronotherapeutic interventions, aligning treatment timing with endogenous biological rhythms to maximize efficacy. For example, administration of antioxidants, mitochondrial protectants, or ischemic preconditioning during phases of high BMAL1/CLOCK activity yields stronger neuroprotection than at opposite circadian phases [113]. Similarly, pharmacological activation of REV-ERB or SIRT1 not only restores clock rhythmicity but also enhances NRF2-mediated antioxidant defense [105,114]. In clinical settings, timed delivery of thrombolytic agents or neuroprotectants based on circadian phase may improve therapeutic outcomes [123,124].

**Figure 4:**
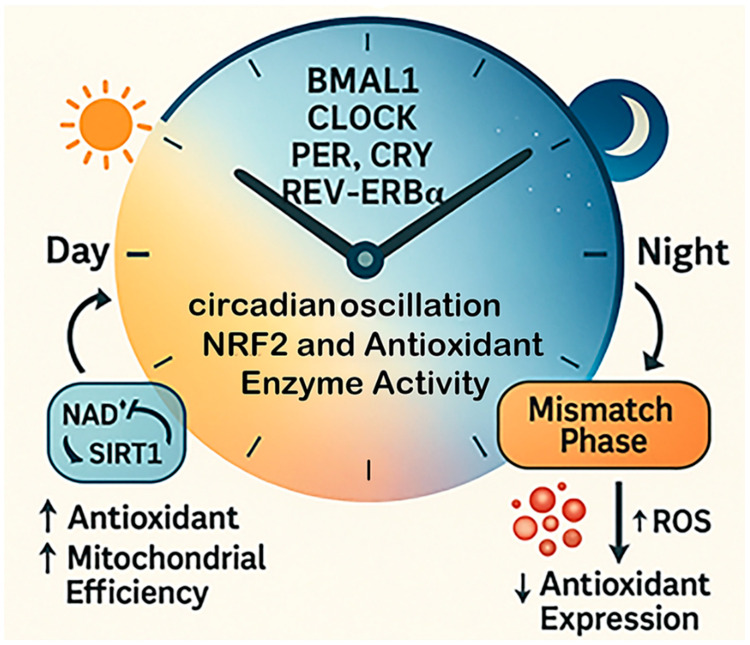
**Temporal redox regulation by circadian rhythms.** This diagram illustrates how circadian rhythms regulate oxidative balance across the 24 h cycle. Core clock genes, including BMAL1, CLOCK, PER, CRY, and REV-ERBα, govern daily fluctuations in antioxidant enzyme activity and mitochondrial redox function. During the active phase, antioxidant expression and mitochondrial efficiency are high, supporting ROS detoxification. During the rest phase, these defenses decline. When circadian alignment is disrupted, the timing of antioxidant production no longer matches ROS generation, leading to redox desynchrony and increased oxidative stress.

Circadian rhythms impose a temporal hierarchy over redox homeostasis, coordinating mitochondrial metabolism, antioxidant defenses, and inflammatory tone with the light–dark cycle. Disruption of this temporal alignment, by aging, metabolic stress, or lifestyle misalignment, creates a state of temporal disorganization that amplifies oxidative injury and worsens ischemic outcomes (Figure 4). Understanding and harnessing circadian redox control thus offer a promising frontier for refining both the timing and the mechanistic targeting of stroke interventions.

## 5. Interplay of Aging, Metabolic Stress, and Circadian Rhythms in Modulating Redox Vulnerability

Oxidative imbalance in the brain seldom results from a single pathogenic trigger; instead, it arises from the convergence of aging, metabolic overload, and circadian disruption, which collectively compromise redox coordination at molecular, cellular, and systemic levels [125,126]. These conditions, while distinct in origin, share overlapping mechanisms involving mitochondrial dysfunction, impaired antioxidant signaling, and inflammatory activation, that amplify each other through reciprocal feedback loops. This integrative framework reframes oxidative stress not merely as a downstream consequence of brain injury but as a primed pathological state, progressively conditioned by the cumulative loss of redox resilience over time. Understanding how these temporal and metabolic dimensions intersect offers critical insight into why certain individuals exhibit heightened susceptibility to ischemic or neurodegenerative insults and reveals new opportunities for targeted intervention across the lifespan.

### 5.1. Convergent Mechanistic Nodes

At the molecular core of aging, metabolic stress, and circadian disruption lie several regulatory hubs that determine the brain’s capacity to sustain redox equilibrium.

**Mitochondrial-NAD^+^ Axis:** Both aging and metabolic overload deplete intracellular NAD^+^ reserves and suppress Sirtuin activity, diminishing the deacetylation of metabolic and antioxidant enzymes [127,128]. Circadian disruption further exacerbates this deficit by desynchronizing Nampt-driven NAD^+^ biosynthesis from cellular energy demand [12,129]. Together, these events result in inefficient oxidative phosphorylation, enhanced electron leakage, and impaired ROS detoxification.

**NRF2-KEAP1 System:** The NRF2-centered antioxidant network represents another shared node of vulnerability. Aging attenuates NRF2 activation, while obesity suppresses its transcription through chronic inflammatory signaling [130,131]. Circadian misalignment further uncouples NRF2 rhythmicity from ROS production cycles, impairing the timely induction of antioxidant genes [132,133]. Collectively, these disturbances blunt the brain’s adaptive redox responses to acute oxidative challenges such as ischemia or reperfusion.

**Inflammatory-Oxidative Coupling:** Microglial priming in aging, adipokine-driven inflammation in obesity, and clock gene disruption converge on persistent NF-κB activation and NADPH oxidase stimulation [134,135,136]. This feed-forward inflammatory–oxidative circuit maintains elevated basal ROS levels and endothelial activation, transforming transient redox fluctuations into sustained pathogenic stress.

**Functional Evidence from Pathway Impairment and Rescue Studies:** Experimental studies directly demonstrate the causal importance of these pathways in stroke outcomes. Genetic or pharmacological impairment of NRF2 signaling leads to insufficient antioxidant induction, increased oxidative damage, blood–brain barrier disruption, and larger infarct volumes following ischemia–reperfusion, whereas restoration of NRF2 activity markedly reduces tissue injury and improves neurological recovery [31,137]. Similarly, suppression of the NAD^+^–SIRT–PGC-1α axis exacerbates mitochondrial dysfunction and oxidative stress, while replenishment of NAD^+^ pools or reactivation of sirtuin signaling preserves mitochondrial integrity, limits infarct expansion, and improves functional outcomes in ischemic models [35,36]. In parallel, loss of AMPK activity removes a critical energy-stress checkpoint, aggravating metabolic failure and redox imbalance during ischemia, whereas AMPK reactivation enhances ischemic tolerance and reduces neuronal injury [138,139]. Overall, these findings establish NRF2, SIRT–PGC-1α, and AMPK pathways as functional determinants of redox flexibility and stroke severity rather than passive correlates.

**Figure 5:**
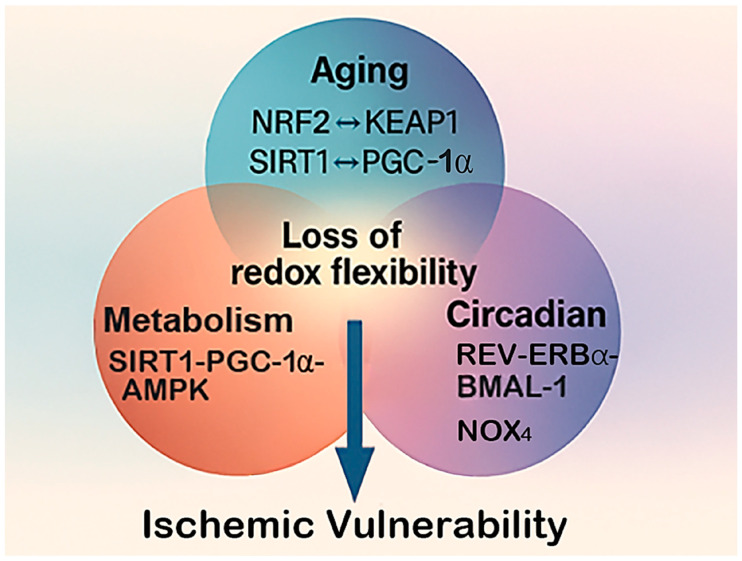
**Converging pathways of redox vulnerability.** This diagram illustrates how aging, metabolic stress, and circadian disruption converge on shared molecular pathways that control oxidative balance in the brain. The central hub represents the loss of redox flexibility, a state in which adaptive antioxidant and metabolic responses become impaired. Key regulatory nodes, such as the NRF2–KEAP1 antioxidant system, the SIRT1–PGC-1α–AMPK energy-redox axis, and NOX4-mediated ROS generation, serve as common points of interaction among these three physiological stressors. Their combined disruption leads to cumulative oxidative burden and heightened ischemic vulnerability.

Together, these mechanistic intersections illustrate how distinct physiological insults converge on common pathways of redox fragility (Figure 5), predisposing the brain to exaggerated oxidative injury when ischemic or inflammatory events occur.

### 5.2. Cellular and Network-Level Interactions

The combined impact of aging, metabolic stress, and circadian disruption extends beyond molecular dysfunction to drive cellular and network-level disturbances that heighten cerebral susceptibility to oxidative damage.

**Neuronal Energy Failure:** Persistent NAD^+^ depletion and mitochondrial inefficiency reduce ATP generation, compromising ion homeostasis and synaptic transmission. In this energy-deficient state, neurons rely increasingly on glycolysis, which further enhances ROS production and lactate accumulation [140,141]. Such chronic metabolic strain predisposes neurons to excitotoxic and apoptotic cascades when ischemia occurs.

**Astrocyte and Microglia Dysregulation:** Under combined metabolic and circadian stress, astrocytes exhibit reduced GSH synthesis and impaired lactate shuttling to neurons, weakening neuroenergetic support [142]. Concurrently, microglia adopt a pro-oxidant, NF-κB-driven phenotype, characterized by exaggerated cytokine and ROS release [143]. The resulting imbalance between neuroprotective and neurotoxic glial responses sustains a chronic low-grade inflammatory tone that sensitizes the brain for acute oxidative injury.

**Endothelial and Network Compromise:** Endothelial cells exposed to persistent oxidative–inflammatory signaling lose NO bioavailability, leading to vascular constriction and impaired microcirculation [144]. Tight junction disruption increases BBB permeability, facilitating leukocyte infiltration and intensifying redox stress within the neurovascular unit [145]. These vascular changes weaken neurovascular coupling and cerebral autoregulation, both essential for ischemic tolerance.

Through these interconnected cellular events, chronic systemic stress evolves into network-level redox instability, rendering the brain hypersensitive to metabolic and ischemic perturbations.

### 5.3. From Preconditioning to Pathology

Under normal conditions, mild and transient oxidative fluctuations driven by circadian metabolism strengthen the brain’s defense capacity by activating antioxidant and repair pathways [13]. Clock proteins such as BMAL1 and CLOCK rhythmically regulate antioxidant enzymes (e.g., SOD, GPx, catalase), while short bursts of ROS during high metabolic activity trigger NRF2-dependent detoxification and repair gene expression, maintaining redox balance and cellular resilience.

In this context, ischemic preconditioning (IPC) represents a well-established physiological example of adaptive redox conditioning. IPC, often initiated by a prior mild or sublethal ischemic episode, induces coordinated protective gene programs that enhance tolerance to subsequent ischemic injury. At the molecular level, IPC activates transcription factors such as NRF2, HIF-1α, and FOXO3, leading to upregulation of antioxidant and cytoprotective genes (HMOX1, SOD2, BCL2), metabolic regulators (PGC-1α, NAMPT), and autophagy-related genes (BECN1, LC3, ATG5) [146,147,148]. In parallel, IPC promotes the release of neurotrophic factors such as BDNF and GDNF, modulates inflammatory signaling by suppressing excessive NF-κB activation, and enhances mitochondrial quality control through improved mitophagy and biogenesis [149,150]. Collectively, these responses limit oxidative damage, preserve mitochondrial function, and increase neuronal survival following subsequent ischemic insults.

However, with aging, obesity, or circadian disruption, this adaptive rhythm breaks down. Oxidative activity becomes unstable and damaging as mitochondrial efficiency, NAD^+^ levels, and antioxidant enzyme activity decline [136,146]. The brain transitions from a protected to a primed-for-injury state, marked by chronically elevated ROS, weakened antioxidant defenses, and persistent inflammation. When ischemia occurs, this pre-existing oxidative burden magnifies the ROS surge during reperfusion, overwhelming detoxification systems and driving irreversible mitochondrial and neuronal damage [17,45]. This framework explains why aged or metabolically compromised individuals experience larger infarcts, slower recovery, and diminished responsiveness to conventional antioxidant therapies once redox homeostasis becomes chronically rigid and maladaptive.

### 5.4. Systemic Integration and Therapeutic Implications

The interplay among aging, metabolic stress, and circadian disruption extends beyond cellular pathology to shape systemic redox homeostasis, influencing vascular function, immune reactivity, and overall brain resilience. These interconnected processes define a continuum in which local oxidative injury reflects broader physiological disorganization.

**Systemic Redox-Inflammatory Crosstalk:** Chronic metabolic inflammation elevates circulating ROS and cytokines that penetrate or signal across the BBB, establishing a pro-oxidant milieu even before cerebral ischemia occurs [147]. Aging further impairs hepatic and renal clearance of oxidative metabolites [148,149], while circadian misalignment desynchronizes hormonal regulators such as cortisol and melatonin that normally modulate systemic redox balance [20,126]. The result is a body-wide oxidative tone that predisposes the brain to exaggerated ischemic cascades.

**Figure 6:**
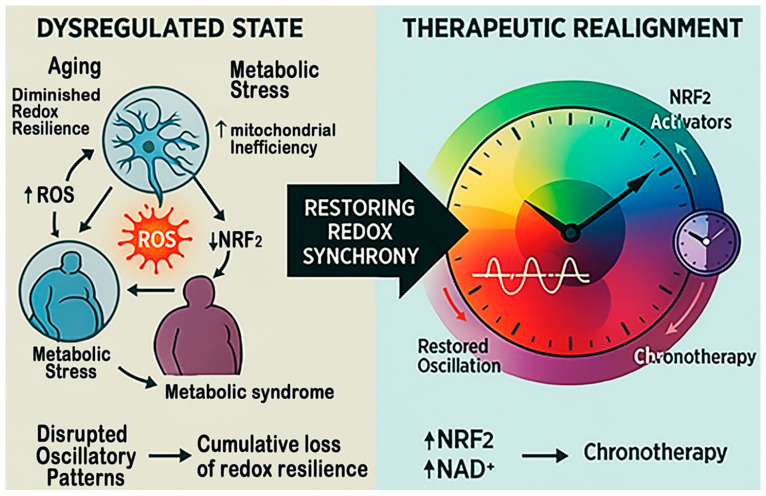
**Restoring redox synchrony for therapeutics in stroke.** This figure compares the dysregulated and restored redox states that shape stroke outcomes. On the left, aging, metabolic stress, and circadian disruption reduce NAD^+^ and SIRT1 activity, suppress NRF2 signaling, and activate NF-κB and NOX4, creating self-reinforcing oxidative and inflammatory loops. On the right, NAD^+^ boosters, NRF2 activators, and chronotherapy restore mitochondrial function, antioxidant transcription, and circadian alignment, thereby re-establishing redox balance and improving resilience to ischemic injury.

**Therapeutic Integration:** Addressing this multidimensional vulnerability requires strategies that target not only oxidative endpoints but also the temporal and metabolic contexts in which they arise. Restoring NAD^+^ homeostasis through precursors (e.g., nicotinamide riboside), enhancing NRF2 signaling, and pharmacologically activating SIRT1 or REV-ERB represent viable molecular approaches. Equally important is the implementation of chronotherapeutic scheduling [110,150], aligning antioxidant, metabolic, or neuroprotective interventions with circadian peaks in endogenous defense capacity (Figure 6).

**Physical Activity as a Systemic Redox Modulator:** Regular physical activity represents a potent, non-pharmacological regulator of systemic redox homeostasis that intersects with aging, metabolic stress, and circadian regulation. Exercise enhances mitochondrial efficiency and antioxidant capacity through activation of AMPK–PGC-1α–SIRT1 signaling, promotes NRF2-dependent antioxidant gene expression, and improves NAD^+^ availability [151,152]. In parallel, physical activity suppresses chronic NOX-driven oxidative stress, improves insulin sensitivity, and reinforces circadian rhythmicity by stabilizing clock gene oscillations [153,154]. Clinically, higher levels of physical activity are associated with reduced stroke incidence, improved vascular function, and enhanced post-stroke recovery, supporting its role in preserving redox flexibility and mitigating ischemic injury [155].

**Integrated Perspective:** Viewing redox imbalance as a network disorder, spanning metabolic, temporal, and inflammatory domains, shifts therapeutic focus from isolated antioxidant supplementation to systemic recalibration of redox regulation. This integrated framework offers a more effective path for improving stroke prevention and recovery by restoring physiological coordination between energy metabolism, circadian timing, and oxidative control.

The aging-metabolic-circadian triad thus represents a unified determinant of cerebral redox stability. Their convergence undermines both the strength and timing of antioxidant defense, creating a chronic state of vulnerability that amplifies ischemic injury. Recognizing oxidative stress as an emergent property of systemic dysregulation, rather than an isolated event, offers a conceptual foundation for time-informed, metabolism-aware neuroprotective strategies.

### 5.5. Therapeutic Interventions That May Disrupt Redox Homeostasis

While many pharmacological treatments are essential for managing cardiovascular and metabolic comorbidities in stroke patients, certain therapeutic strategies may inadvertently promote chronic redox imbalance when used chronically. Prolonged exposure to high-dose antioxidants, for example, may suppress physiological redox signaling and impair adaptive antioxidant responses, potentially reducing endogenous resilience to ischemic stress [156]. Similarly, sustained inhibition of mitochondrial respiration or excessive activation of oxidative pathways by some chemotherapeutic or immunosuppressive agents can exacerbate basal ROS production and mitochondrial dysfunction [157].

Metabolic interventions that disrupt NAD^+^ homeostasis or blunt AMPK and SIRT1 signaling, such as chronic overuse of certain sedatives, anticholinergic agents, or metabolic suppressors, may further compromise redox flexibility [158], particularly in aged or metabolically stressed individuals. In addition, pharmacological agents that perturb circadian organization, including long-term glucocorticoid therapy or sleep-disrupting medications [159], can desynchronize antioxidant defense from metabolic demand, amplifying oxidative vulnerability.

These observations underscore the importance of considering redox timing, metabolic context, and circadian alignment when selecting or optimizing therapeutic regimens in patients at risk for stroke. Avoiding or carefully managing treatments that induce persistent oxidative imbalance may help preserve redox adaptability and improve neurological outcomes.

## 6. Conclusions and Future Perspectives

The brain’s oxidative landscape is not a static battlefield but a dynamic continuum shaped by circadian timing, metabolic demands, and systemic health. This review integrates evidence showing that redox fluctuations, once considered purely detrimental, serve as temporal signals that calibrate cellular defense and repair. However, when aging, metabolic dysregulation, or circadian disruption distort these rhythms, adaptive oxidative cycles collapse into chronic imbalance, predisposing the brain to ischemic vulnerability.

Future research should therefore prioritize multi-scale investigations that bridge molecular, cellular, and systemic levels, linking mitochondrial bioenergetics, NAD^+^ metabolism, and clock gene expression with functional outcomes after brain injury.

Understanding this transformation from rhythmic regulation to redox rigidity reframes oxidative stress as an emergent property of systemic desynchronization rather than an isolated molecular event. Despite growing mechanistic insight, major knowledge gaps remain. In particular, how redox oscillations are coordinated across different brain cell types, how systemic metabolic and circadian signals are integrated at the neurovascular unit, and how these dynamics differ across aging and disease states remain poorly understood [160]. In addition, most experimental studies capture redox signaling at single time points, limiting our ability to resolve temporal causality and adaptive versus maladaptive oxidative responses [161].

Future research should therefore prioritize time-resolved and multi-scale approaches that link mitochondrial bioenergetics, NAD^+^ metabolism, and clock gene activity to functional outcomes after ischemic injury. Longitudinal studies combining redox profiling with circadian phase, metabolic status, and cell-type-specific responses will be essential for defining windows of vulnerability and resilience. Translational efforts are also needed to determine whether redox rhythmicity can be reliably assessed and targeted in clinical populations.

Therapeutically, interventions should move beyond nonspecific antioxidant supplementation toward precision redox modulation, strategies that restore the timing, localization, and amplitude of oxidative signaling. Promising approaches include pharmacological targeting of NRF2, SIRT1, and REV-ERB pathways, reinforcement of circadian alignment, and metabolic repletion through NAD^+^ precursors. Incorporating chronotherapeutic principles into neuroprotective treatment design may further optimize therapeutic windows following stroke or brain injury. In parallel with pharmacological strategies, lifestyle interventions such as regular physical activity may play a critical role in restoring redox synchrony and enhancing resilience to ischemic injury.

Ultimately, embracing the temporal dimension of redox biology may transform how neurodegenerative and ischemic diseases are understood and treated, shifting the paradigm from reactive damage control to proactive, rhythm-based restoration of brain resilience.

## Data Availability

No new data were created or analyzed in this study. Data sharing is not applicable to this article.
